# Letter to the editor of heliyon re: The predicting value of the ratio of levator hiatus diameter to fetal head circumference in pregnant women at 37 weeks of gestation in the progression of the second stage of labor and levator ani injury 6 weeks postpartum”

**DOI:** 10.1016/j.heliyon.2024.e31361

**Published:** 2024-05-16

**Authors:** James Ashton-Miller, John DeLancey

**Affiliations:** Ann Arbor, USA; Ann Arbor, USA

Sir

In their paper Dr. Gan and colleagues [1] used intrapartum ultrasound scanning to test the very reasonable hypothesis that the ratio of levator hiatus diameter to head circumference might be used to predict the duration of the second stage of labor as well as the risk for levator ani injury. Simply put, when a large fetal head must be pushed through a smaller hiatus, one might expect that this might be associated with a longer second stage of labor as well as a higher risk for levator injury. Their results confirm this idea. This is a useful contribution to the literature on this topic because of its potential for use as a screening tool. In the future one might be able to use it to minimize the risk of long second stages as well as the risk for levator and hiatal injuries.

But this idea is not new. In their Introduction reviewing the literature, the authors fail to acknowledge the prior ultrasound study of Rostaminia et al. [2] who were the first to attempt such predictions. A weakness of the Rostaminia et al. approach in terms of clinical applicability was the fact that they used postpartum measures of head circumference rather than prepartum measures to test the ratio. In a study published the same year, Tracy et al. [3] developed and tested what they called the “Capacity-Demand” ratio - the very same ratio being tested by Gan et al. In that paper they laid out the theoretical framework for the ratio, with the “capacity” quantifying the size of the mother's outlet and the “demand” the size of the baby's head. When the capacity is very much larger than the demand the birth can proceed easily. When it is close to the demand, the outcome is uncertain. When the capacity is much less than the demand, a longer second stage can be expected, and a vacuum/forceps intervention will be necessary. They directly tested that idea using a computer model based on the published variations in the two parameters constituting the Capacity-Demand ratio. They used a similar approach for the risk for risk of levator injury which they refined in a follow-up paper, Tracy et al. [4] by adding actual measures of distal maternal birth canal tissue elasticity and viscosity during the first stage of labor to predict the length of the active second stage of labor and risk for levator injury, both goals of the Gan et al. paper.

It is incumbent upon any authors to exercise due diligence and find the earlier papers addressing the central problem addressed in their paper. These are usually summarized briefly in the Introduction of the paper. It would have been normal scientific etiquette for Gan et al. to have acknowledged the papers most relevant to the central idea tested in their paper, namely the “Capacity-Demand” ratio as a prediction tool: those papers are easily found in a Google search using key words like “levator hiatus fetal head prediction”, including the three papers mentioned above. It matters not that the Rostaminia et al. paper employed an ultrasound method to measure one of the capacity-demand variables and a physical method to measure the other, or that the Tracy et al. papers employed morphological, biomechanical and *in vivo* tissue property data in predictive computer models. All three tested the central idea of the predictive ability of the capacity-demand ratio six or more years prior to Gan et al. Finally, in the Discussion of a scientific paper it is usual practice to consider how the results in that paper corroborate and extend findings already published in the literature on the central focus of the paper, a discussion that again is missing in the Gan et al. paper because the most salient papers were never cited.

## Funding statement

This research did not receive any specific grant from funding agencies in the public, commercial, or not-for-profit sectors.

## Data availability statement

No data were used for the research described in the article.

## CRediT authorship contribution statement

**James Ashton-Miller:** Writing – review & editing, Writing – original draft, Conceptualization. **John DeLancey:** Writing – review & editing, Writing – original draft.

## Declaration of competing interest

The authors received 10.13039/100007197United States Public Health Service funding in the past three years in the form of a sub-contract to their institution from grant R44 HD096987 to Maternal Medical, LLC to support their studies of biomechanical data from a device designed to pre-stretch the birth canal in the first stage of labor.
